# Lipoxin A4 Ameliorates Acute Pancreatitis-Associated Acute Lung Injury through the Antioxidative and Anti-Inflammatory Effects of the Nrf2 Pathway

**DOI:** 10.1155/2019/2197017

**Published:** 2019-11-06

**Authors:** Wen Ye, Chenlei Zheng, Dinglai Yu, Fan Zhang, Reguang Pan, Xiaofeng Ni, Zhehao Shi, Zhongjing Zhang, Yukai Xiang, Hongwei Sun, Keqing Shi, Bicheng Chen, Qiyu Zhang, Mengtao Zhou

**Affiliations:** ^1^Key Laboratory of Diagnosis and Treatment of Severe Hepato-Pancreatic Diseases of Zhejiang Province, The First Affiliated Hospital, Wenzhou Medical University, Wenzhou, 325015 Zhejiang Province, China; ^2^Department of Surgery, The First Affiliated Hospital, Wenzhou Medical University, Wenzhou, 325015 Zhejiang Province, China; ^3^The Pancreatitis Treatment Center, The First Affiliated Hospital, Wenzhou Medical University, Wenzhou, 325015 Zhejiang Province, China

## Abstract

Acute lung injury (ALI) is a critical event involved in the pathophysiological process of acute pancreatitis (AP). Many methods have been widely used for the treatment of AP-ALI, but few are useful during early inflammation. Lipoxin A4 (LXA4), a potent available anti-inflammatory and novel antioxidant mediator, has been extensively studied in AP-ALI, but its underlying mechanism as a protective mediator is not clear. This research was conducted to identify the possible targets and mechanisms involved in the anti-AP-ALI effect of LXA4. First, we confirmed that LXA4 strongly inhibited AP-ALI in mice. Next, using ELISA, PCR, and fluorescence detection to evaluate different parameters, LXA4 was shown to reduce the inflammatory cytokine production induced by AP and block reactive oxygen species (ROS) generation in vivo and in vitro. In addition, TNF-*α* treatment activated the nuclear factor E2-related factor 2 (Nrf2) signaling pathway and its downstream gene heme oxygenase-1 (HO-1) in human pulmonary microvascular endothelial cells (HPMECs), and LXA4 further promoted their expression. This study also provided evidence that LXA4 phosphorylates Ser40 and triggers its nuclear translocation to activate Nrf2. Moreover, when Nrf2-knockout (Nrf2^−/−^) mice and cells were used to further assess the effect of the Nrf2/HO-1 pathway, we found that Nrf2 expression knockdown partially eliminated the effect of LXA4 on the reductions in inflammatory factor levels while abrogating the inhibitory effect of LXA4 on the ROS generation stimulated by AP-ALI. Overall, LXA4 attenuated the resolution of AP-induced inflammation and ROS generation to mitigate ALI, perhaps by modulating the Nrf2/HO-1 pathway. These findings have laid a foundation for the treatment of AP-ALI.

## 1. Introduction

Acute pancreatitis (AP) is an inflammatory process characterized by local and systemic inflammatory response syndrome (SIRS) and high morbidity and mortality due to the activation of pancreatic zymogen, leading to autodigestion of pancreatic acinar cells [1, 2]. SIRS and multiorgan dysfunction syndrome (MODS) follow after damage to pancreatic acinar cells. Most patients with AP experience a mild disease course, but 10-20% develop severe AP (SAP), which is a life-threatening condition [3, 4]. The lung is the most common susceptible organ. The primary cause of mortality in early-stage AP patients is frequently associated with acute lung injury (ALI) and acute respiratory distress syndrome (ARDS) [5, 6]. Therefore, aggressive early intervention and optimized therapy for AP-ALI are very important to improve outcomes.

Several studies have demonstrated that the accumulation of a large number of neutrophils in the lungs is a common pathophysiological feature of ALI and that enhanced generation of reactive oxygen species (ROS) and increased production of proinflammatory cytokines play a crucial role in acute lung damage [7–10]. Therefore, AP-ALI can be attenuated by either inhibiting the generation of ROS or scavenging inflammatory factors such as TNF-*α*, IL-1*β*, IL-6, and E-selectin.

Nuclear factor erythroid 2-related factor-2 (Nrf2) combined with a promoter antioxidant response element (ARE) is the key regulator of intracellular antioxidant enzymes and thus participates in eliminating ROS and preventing damage to cells caused by oxidative stress [11–13]. The Nrf2/ARE signaling pathway constitutes a pleiotropic cellular defense, and heme oxygenase-1 (HO-1) is one of the most important antioxidant enzymes triggered in this signaling pathway [12], which has received widespread attention in recent years due to its anti-inflammatory effects [14, 15].

Although many studies have focused on developing new clinical therapies for ALI and respiratory insufficiency, no particularly effective drugs are currently available beyond routine life-support treatment. Lipoxin A4 (LXA4), one of the most important physiological lipoxins, is a member of the family of endogenous anti-inflammatory molecules generated from arachidonic acid during the onset of the inflammatory response [16]. Notably, studies have demonstrated that LXA4 can play a role in blocking the generation of proinflammatory cytokines and preventing oxidative stress-mediated tissue injury [17, 18]. However, the mechanism of protection has not yet been entirely elucidated.

In the present study, we explored whether the administration of LXA4 can protect lung tissue against oxidative and proinflammatory molecule-mediated injury as well as whether LXA4 exerts beneficial effects on AP-ALI by activating the Nrf2/HO-1 pathway.

## 2. Materials and Methods

### 2.1. Animals

C57BL/6 wild-type (WT) mice were obtained from the Laboratory Animal Center of Wenzhou Medical University (Wenzhou, CN). Nrf2-knockout (Nrf2^−/−^) mice were obtained from the Jackson Laboratory (Bar Harbor, ME, USA) and bred by the Institute of Model Animals (Nanjing, CN) [19]. Offspring were bred with Nrf2^−/−^ male and female mice, and genotypes were identified by PCR before the experiment. All the mice used in the present research were 6-week-old male mice weighing 20-25 g, and the experiments were performed in full compliance with the guidelines of the Institutional Animal Committee of Wenzhou Medical University. WT and knockout animals were randomly assigned to three experimental groups: the control group (control)—0.9% saline (ip); the AP group (AP)—caerulein (50 *μ*g/kg, 7 times, ip)+LPS (10 mg/kg, ip); and the AP+LXA4 group (AP+LXA4)—LXA4 (0.1 mg/kg, ip)+caerulein (50 *μ*g/kg, 7 times, ip)+LPS (10 mg/kg, ip). Each group included six mice. All animals were killed 24 h after drug injection.

### 2.2. Histopathological Analysis

Pancreatic and pulmonary tissue samples were collected and immediately fixed in paraformaldehyde for 48 h, followed by dehydration with graded alcohol and embedding in paraffin. The tissues were semiserially cut into 5 *μ*m sections, and the tissue samples were deparaffinized twice for 15 min using xylene and rehydrated for 5 min, respectively, in 100, 95, 90, 80, and 75% ethanol. Then, the sections were stained with hematoxylin-eosin (HE; G1120, Solaibio, CN). After observation under a light microscope, pancreatic pathological scores and lung injury scores were assessed as previously described [20, 21]. Each slide was divided into five equal areas at 400x magnification to estimate the histopathological alterations. If discrepancies arose between the two examiners, a conclusive agreement was reached with the involvement of the third investigator.

### 2.3. Amylase Activity Estimation

Serum amylase activity in the groups treated with different solutions was quantified using iodine-amylum colorimetry following the manufacturer's protocol (1530511002, Jon Ln, CN).

### 2.4. Wet-to-Dry Weight (*W*/*D*) Ratios

The right lung was excised and blotted with a filter paper to absorb the surface water. Then, its weight was measured on an electronic scale and recorded as the wet weight (*W*). The right lung was then dried at 70°C in an oven for 48-72 h, and its dry weight (*D*) was measured until the weight was constant. The *W*/*D* ratio was calculated using the following formula: *W*/*D* = (wet weight − dry weight)/dry weight.

### 2.5. ELISA Quantification

The serum levels of TNF-*α*, IL-1*β*, and E-selectin in mice were determined by ELISA using quantikine ELISA kits according to the manufacturer's instructions (ab208348, ab100704, and ab201279; Abcam, USA).

### 2.6. Immunohistochemical (IHC) Analysis

Immunohistochemistry was used to qualitatively analyze the expression of phenotypic markers. To examine the expression of IL-6 in lung tissue samples, an IHC examination was carried out with formalin-fixed, paraffin-embedded sections (4 *μ*m) mounted on adhesion microscope slides (80312-3161, Citotest, CN). The sections were deparaffinized and rehydrated using xylene and graded concentrations of alcohol. The samples were boiled in antigen retrieval buffer containing citrate-hydrochloric acid (0.01 mol/l) using a microwave unit at full power for 15 min. Then, blocking with hydrogen peroxide (3% H_2_O_2_) was performed for 10 min to reduce the influence of endogenous peroxidases. Nonspecific proteins were blocked with 5% donkey serum at 37°C for 1 h. The sections were incubated with an anti-IL-6 antibody (1 : 500, ab6672, Abcam, USA) overnight at 4°C. The sections were further processed by an indirect immunoperoxidase technique using secondary antibodies, and the immunocomplexes were visualized as a brown color with diaminobenzidine (DAB, P0202, Beyotime, CN). Negative control sections were processed similarly, but the primary antibody was replaced with the antibody diluent. The slides were stained with Harris hematoxylin (G1120, Solaibio, CN). Representative images were captured with a microscope (Leica, Jena, GER).

### 2.7. Flow Cytometry

Lung tissue samples were cut into pieces, continuously agitated, and gently ground to extract cells, and ROS were quantified by the ROS-sensitive fluorophore DCFH-DA using the ROS Assay Kit (S0033, Beyotime, CN) according to the manufacturer's protocol [22, 23]. Then, cells from the lung tissue were incubated with DCFH-DA and analyzed for fluorescence intensity using a FACSCalibur flow cytometer (BD Biosciences, USA). The data were analyzed with FlowJo (version 10.0.7).

### 2.8. Cell Culture

Human pulmonary microvascular endothelial cells (HPMECs) were obtained from ScienCell Research Laboratories (San Diego, CA, USA). Cells were grown in endothelial cell medium (1001, ScienCell Research Laboratories, USA) containing 10% fetal bovine serum (0025, ScienCell Research Laboratories, USA), endothelial cell growth supplement (1052, ScienCell Research Laboratories, USA) and 5 ml of penicillin/streptomycin solution (0503, ScienCell Research Laboratories, USA). Experiments were performed when the HPMECs reached 80%-90% confluence.

### 2.9. RNA Extraction and qRT-PCR

RNA was extracted from HPMECs by Trizol Reagent (15596026, Thermo Fisher Scientific, USA) according to the manufacturer's instructions. The RNA expression of IL-1*β*, IL-6, and E-selectin was detected by RT-PCR with mRNA-specific primers (Sangon Biotech, CN). One microgram of RNA was reverse-transcribed into cDNA using the Reverse Transcription System (4374966, Thermo Fisher Scientific, USA). RT-PCR was performed with SYBR Green Supermix with ROX (A25742, Thermo Fisher Scientific, UK) using a PCR detection system (7500fast, Applied Biosystems, CA). Ct values were used to quantify the mRNA levels, and the Ct value of each target mRNA was standardized to the Ct value of *β*-actin using the 2^-*ΔΔ*Ct^ method. The primer pairs used are listed in Table 1.

### 2.10. Measurement of Intracellular ROS Levels

Intracellular levels of ROS were quantified with dehydroergosterol (DHE) (S0063, Beyotime, CN) according to the manufacturer's recommendation [24, 25]. HPMECs were seeded in 6-well plates and incubated with drugs. Then, they were incubated with DHE for 15 min, and the fluorescence intensity was observed using a Nikon Eclipse TI fluorescence microscope (Nikon Corporation, Tokyo, JP). Next, trypsinization was used to collect the cells, which were resuspended in PBS. To detect fluorescence, a C6 flow cytometer (BD Biosciences, USA) was used.

### 2.11. Western Blot (WB) Analysis

HPMECs were washed with phosphate-buffered saline (PBS) (20012500BT, Gibco, Thermo Fisher Scientific, USA). Then, the cells were lysed with radio immunoprecipitation assay (RIPA) buffer (P0013B, Beyotime, CN) containing protease (ST506, Beyotime, CN) and phosphatase inhibitors for 20 min. The lysates were then sonicated and centrifuged at 12,000 rpm for 15 min at 4°C to obtain the total protein supernatant. Protein concentrations were determined by a commercial BCA kit (P0010, Beyotime, CN). The samples were denatured for 5 min by heating at 95°C. *β*-Actin was used as the reference for the total cell proteins, and Lamin B was used as the reference for the nuclear fractions. Equal amounts of protein were separated by SDS-PAGE, transferred to PVDF membranes, and blocked with 5% skim milk for 2 h on a rocker. The membranes were then incubated with a primary antibody overnight at 4°C. The following primary antibodies were used: an anti-Nrf2 (ab62352, Abcam, USA), anti-Nrf2 (phosphoS40) (ab76026, Abcam, USA), anti-HO-1 (ab13243, Abcam, USA), anti-*β*-actin (ab76026, Abcam, USA), and anti-Lamin B (ab151735, Abcam, USA). The membranes were then incubated with the appropriate secondary antibodies (Biosharp, CN) for 1 h. The immunoreactive bands were detected by chemiluminescence methods, and densitometry analysis was performed using VisionWorks imaging software (Eastman Kodak Company, Rochester, NY).

### 2.12. Nuclear Protein Extraction

Nuclear fractions were extracted from HPMECs using a nuclear protein extraction kit (P0028, Beyotime, CN) according to the manufacturer's instructions.

### 2.13. Immunofluorescence

HPMECs were seeded at a density of 100,000 cells/well on 20 mm glass coverslips (WHB-12-CS, WHB, CN) and grown to 80% confluence. The cells were incubated with 4% paraformaldehyde for 15 min and permeabilized for 30 min at 37°C with 0.1% Triton X-100 (T8200, Solarbio Life Sciences, CN). Then, the cells were blocked with PBS containing 5% donkey serum for 1 h. The cells were then treated with the anti-Nrf2 antibody overnight at 4°C. The coverslips were incubated for 2 h with an Alexa Fluor 488-conjugated AffiniPure Goat Anti-Rabbit IgG (33106ES60, YEASEN, CN) secondary antibody in the dark. Then, the cells were counterstained with DAPI (36308ES20, YEASEN, CN) for 10 min. The coverslips were visualized using a laser confocal microscope (Leica TCS SP8, Jena, GER).

### 2.14. Transient Transfection and Luciferase Assay

Based on the binding sites KB, ARE, and TRE in the HO-1 protein gene promoter sequence targeted by the AP-1, NF-*κ*B, and Nrf2 pathways, we constructed gene silencing plasmids. HPMECs were cotransfected with the AP-1, NF-*κ*B, or Nrf2 and AP-1+NF-*κ*B plasmids using Lipofectamine 3000. Luciferase activity was measured using a microplate reader (Tecan, Männedorf, CH).

### 2.15. Lentiviral Transfection

Nrf2 lentivirus and negative control lentivirus were chemically synthesized by GeneChem (Shanghai, CN). The lentiviruses were transfected into HPMECs according to the manufacturer's instructions. Nrf2 mRNA expression was detected 72 h later, and Nrf2 expression knockdown cells were used to evaluate the generation of inflammatory factors and ROS.

### 2.16. Statistical Analysis

Values are presented as the mean ± standard deviation (SD). Statistical analysis was performed using GraphPad Prism 7.0 (GraphPad Software, San Diego, CA). Comparisons between two groups were performed by Student's *t*-tests, and Fisher's exact tests were used to determine differences among more than two groups. The results were calculated using data from three independent experiments. *P* < 0.05 was considered statistically significant.

## 3. Results

### 3.1. LXA4 Propagates the Resolution of AP and the Associated ALI Inflammation

**Figure 1:**
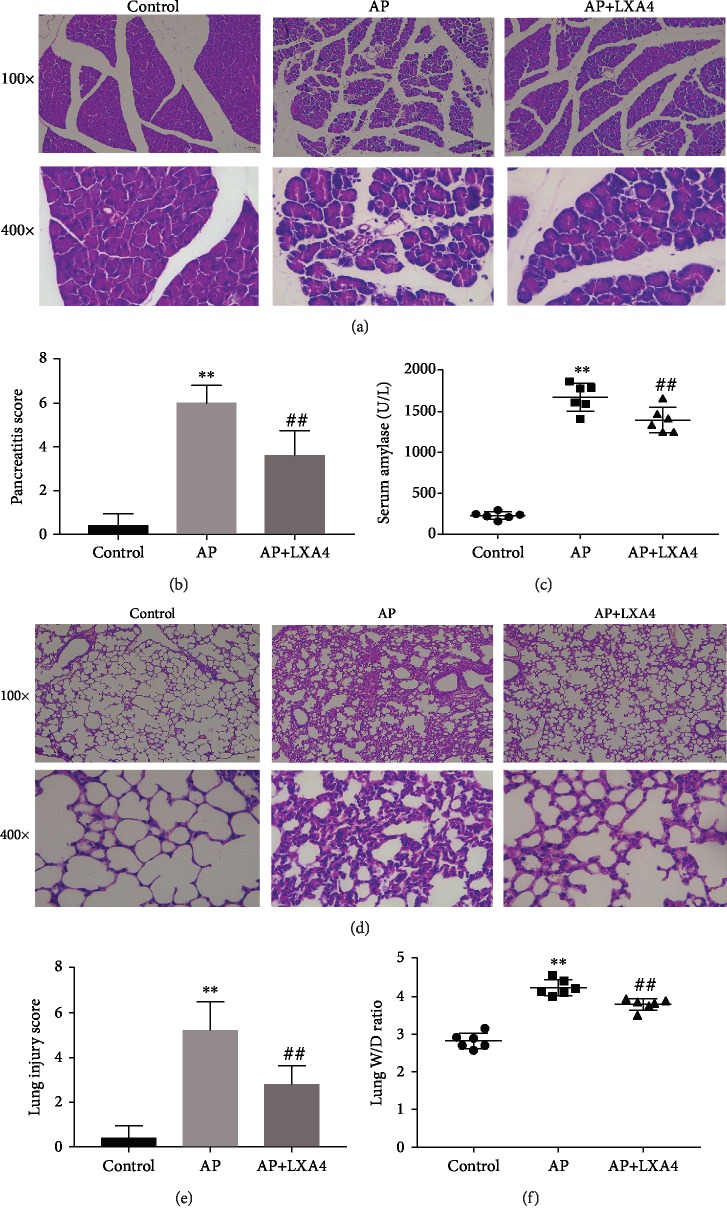
LXA4 effectively inhibited pancreatitis and AP-ALI in mice. Animals were randomly divided into three experimental groups: the AP group (AP) treated with caerulein (50 *μ*g/kg, 7 times, ip)+LPS (10 mg/kg, ip); the AP+LXA4 group treated with LXA4 (0.1 mg/kg, ip)+caerulein+LPS; and the control group treated with 0.9% saline (ip). (a) Representative pathological images of the pancreas (100x and 400x) and (b) histological scores were obtained to evaluate the degree of injury. Slides were evaluated by two independent investigators in a blinded manner. (c) Serum amylase levels were measured by means of iodine-amylum colorimetry. (d) Representative pathological images of the lung (100x and 400x), (e) lung injury scores, and (f) the lung wet/dry weight ratio are shown. ^∗∗^*P* < 0.01 vs. the control group. ^##^*P* < 0.01 vs. the AP group. AP: the acute pancreatitis group; AP+LXA4, the acute pancreatitis+Lipoxin A4 group; AP-ALI: the acute pancreatitis-induced acute lung injury; LPS: lipopolysaccharide.

Samples were stained with HE and observed under an optical microscope. Histological evaluation of the pancreatic tissue showed acinar cell vacuolation, interstitial tissue edema, inflammatory cell infiltration, hemorrhage, and necrosis in the AP group, indicating the successful induction of pancreatitis [26]. Almost no obvious pathological changes were noted in the control group. Compared with the AP group, the AP+LXA4 group exhibited reduced pathology (Figure 1(a)). Pancreatitis severity was estimated by measuring the pathology score. Compared with that in the AP group, the histological score of the pancreas in the AP+LXA4 group was largely attenuated (Figure 1(b)). The levels of amylase were significantly increased in the AP group, whereas they were slightly decreased in the AP+LXA4 treatment group (Figure 1(c)). As shown in Figure 1(d), the control group showed normal pulmonary architecture, while the AP group exhibited an increased alveolar septum thickness caused by alveolar collapse, multiple inflammatory cell infiltrates, and hyperemia in the pulmonary architecture, and the LXA4 treatment group showed an obvious improvement in pulmonary architecture. Histological analyses were performed, and the W/D ratio of the lungs was calculated. Lung injury scores of the AP+LXA4 group were less severe than those of the AP group (Figure 1(e)). The lung W/D ratio results agreed with the lung injury scores (Figure 1(f)). These results indicated that LXA4 administration can attenuate injury to the pancreas and lungs.

### 3.2. LXA4 Attenuates the Levels of Proinflammatory Cytokines and Oxidative Stress in Lung Tissue

**Figure 2:**
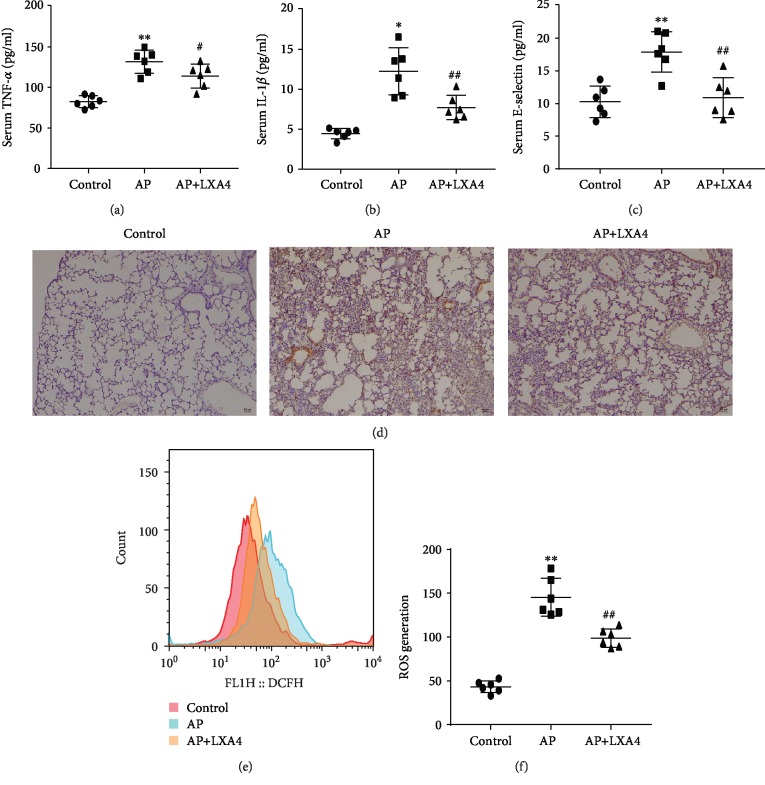
LXA4 inhibited proinflammatory cytokine and ROS generation in AP-ALI mice. Mouse serum and lung tissue samples were collected to evaluate injury 24 h after modeling. (a–c) The serum levels of the inflammatory factors TNF-*α*, IL-1*β*, and E-selectin were measured by ELISA. (d) Immunohistochemistry micrographs of lung tissue sections show the expression of IL-6. (e) Flow cytometric analysis of the cellular ROS in lung tissues in different experimental mouse groups was performed. (f) The quantitative mean fluorescence intensity in each treatment group is shown. ^∗^*P* < 0.05 and ^∗∗^*P* < 0.01 vs. the control group. ^#^*P* < 0.05 and ^##^*P* < 0.01 vs. the AP group. AP: the acute pancreatitis group; AP+LXA4: the acute pancreatitis+Lipoxin A4 group; AP-ALI: the acute pancreatitis-induced acute lung injury; ROS: reactive oxygen species.

As shown by the results of the ELISA analysis in Figures 2(a)–2(c), TNF-*α*, IL-1*β*, and E-selectin levels in serum were significantly increased in the AP group compared with those in the control group. These proinflammatory cytokine levels were reduced by LXA4 treatment. Examples of lung tissue sections stained for IL-6 are shown in Figure 2(d). The results of IHC analyses indicated that IL-6 levels were markedly increased in the AP group. When treated with LXA4, mice showed alleviated damage with weaker IL-6 staining in the lung tissue. Then, we detected the level of ROS in lung tissue homogenates. As shown in Figure 2(e), compared with the control group, the levels of ROS in the AP and AP+LXA4 groups were dramatically enhanced. LXA4 reduced the level of ROS compared with that in the AP group. The numerical statistical histogram is shown in Figure 2(f). The administration of LXA4 markedly reduced the production of inflammatory factors and ROS, thereby inhibiting AP-ALI.

### 3.3. The Inhibitory Effect of LXA4 on HPMEC Inflammation and the Redox State

**Figure 3:**
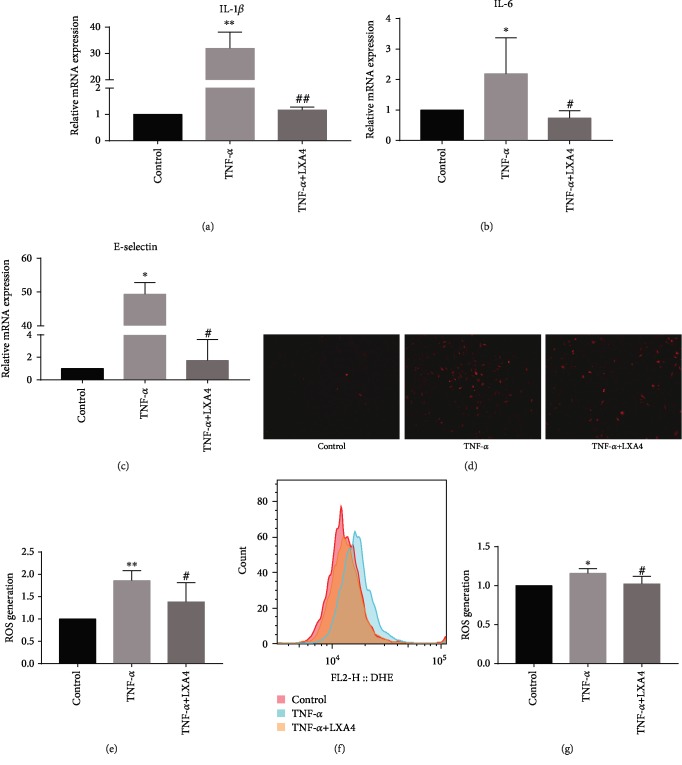
LXA4 alleviated TNF-*α*-induced damage and inhibited ROS generation in vitro. TNF-*α* has been commonly used for AP-ALI experimental modeling in vitro, and we used the concentration established in our previous study. HPMECs were pretreated with 50 ng/ml LXA4 for 1 h, followed by stimulation with 50 ng/ml TNF-*α* for 24 h. (a–c) Fold changes in the mRNA expression of IL-1*β*, IL-6, and E-selectin in HPMECs were calculated. The mRNA levels of IL-1*β*, IL-6, and E-selectin were measured by RT-PCR. (d) Cellular ROS were observed by fluorescence microscopy. (e) The quantitative mean fluorescence intensity in each treatment group is shown. (f) The levels of ROS were determined by flow cytometric analysis. (g) Flow cytometric analysis results are presented as a histogram. ^∗^*P* < 0.05 and ^∗∗^*P* < 0.01 vs. the control group. ^#^*P* < 0.05 and ^##^*P* < 0.01 vs. the TNF-*α* group. TNF-*α*: the TNF-*α* group; TNF-*α*+LXA4: the TNF-*α*+Lipoxin A4 group; ROS: reactive oxygen species; AP-ALI: the acute pancreatitis-induced acute lung injury; HPMECs: human pulmonary microvascular endothelial cells.

To validate our findings in vivo, we established an inflammatory model in HPMECs stimulated with TNF-*α*. As shown in Figures 3(a)–3(c), the RT-PCR results demonstrated that TNF-*α* treatment obviously induced the expression of IL-1*β*, IL-6, and E-selectin at the mRNA level, while LXA4 attenuated this induction in HPMECs. Next, the effect of LXA4 on the redox state of HPMECs under oxidative stress was explored. Fluorescence images (Figures 3(d) and 3(e)) stained with DHE to detect ROS revealed that compared with control treatment, TNF-*α* treatment resulted in a large increase in ROS. LXA4 inhibited the effect of TNF-*α* on ROS generation to some degree. As shown in Figures 3(f) and 3(g), the flow cytometric analysis verified these results. These data indicated that LXA4 may play a role in protecting HPMECs from inflammation and oxidative stress.

### 3.4. LXA4 Promotes Nrf2/HO-1 Pathway Activation in HPMECs

**Figure 4:**
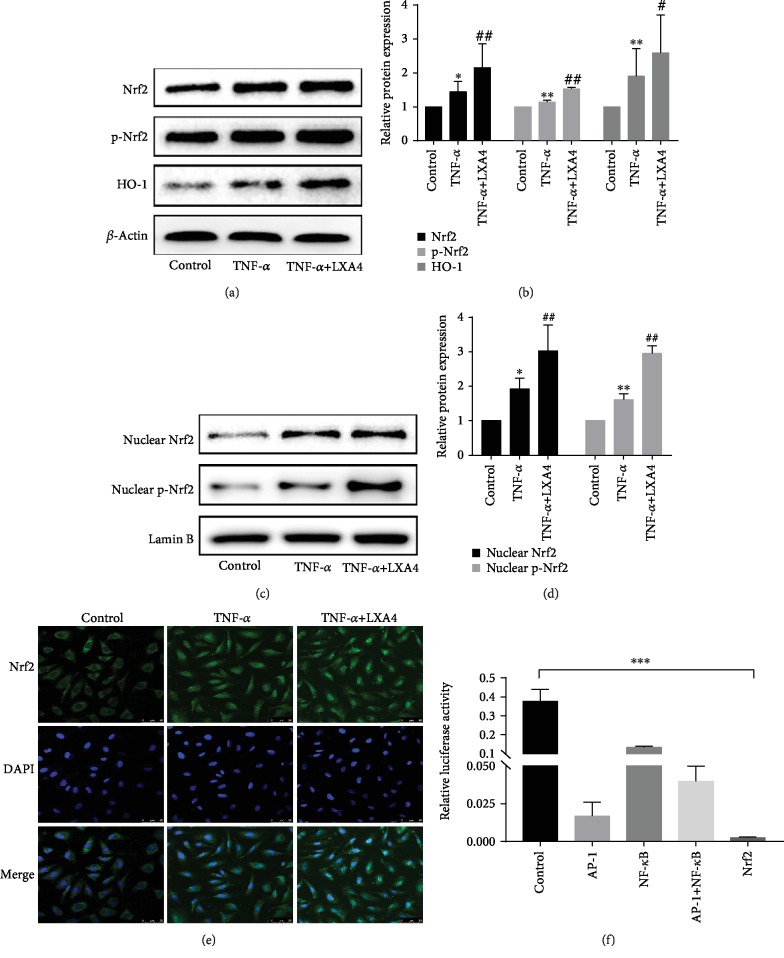
LXA4 activated the Nrf2 pathway and upregulated HO-1 expression. (a) The total protein levels of Nrf2, pSer40-Nrf2, and HO-1 in HPMECs were determined by Western blotting. *β*-Actin was used as an internal control. (b) Relative band densities were quantified, and the normalized values are indicated in the histogram. (c) The nuclear Nrf2 and pSer40-Nrf2 levels were assessed by Western blot analysis. For the internal control, Lamin B was used. (d) The blots were analyzed by densitometry, and the results are expressed in the histogram. (e) Nrf2 nuclear translocation in HPMECs was determined using immunofluorescence analysis. (f) A luciferase reporter assay showed that Nrf2 was the gene that most effectively regulated HO-1. All experiments were performed at least three times. ^∗^*P* < 0.05, ^∗∗^*P* < 0.01 and ^∗∗∗^*P* < 0.001 vs. the control group. #*P* < 0.05 and ##*P* < 0.01 vs. the TNF-*α* group. TNF-*α*: the TNF-*α* group; TNF-*α*+LXA4, the TNF-*α*+Lipoxin A4 group; Nrf2: the nuclear factor E2-related factor 2; p-Nrf2: phosphorylated Nrf2; HO-1: heme oxygenase-1; AP-1: activator protein-1; NF-*κ*B: nuclear factor kappa B.

The Nrf2 pathway is a pathway closely related to oxidative stress and inflammatory responses [27–29]. To test the activation of Nrf2 in HPMECs, we detected the expression of Nrf2 and pSer40 Nrf2 by Western blotting. As shown in Figures 4(a) and 4(b), the expression levels of Nrf2 and pSer40 Nrf2 were significantly upregulated after TNF-*α* treatment. Moreover, the expression levels in the TNF-*α*+LXA4 group were even higher than those in the other two groups. These results suggested that Nrf2 can be activated by phosphorylation at Ser40. To further understand the molecular mechanisms underlying the effect of LXA4 on Nrf2, we performed WB and immunofluorescence experiments using HPMECs. TNF-*α* treatment increased the Nrf2 protein level in the nucleus. In addition, when LXA4 was administered simultaneously, the nuclear Nrf2 level was even higher (Figures 4(c) and 4(d)). The effect of LXA4 on Nrf2 activation was further demonstrated by confocal microscopy. As demonstrated by the results of the immunofluorescence analysis shown in Figure 4(e), weak and diffuse Nrf2 staining in the cytoplasm was observed in the control group. Following TNF-*α* treatment, Nrf2 was activated and translocated into the cell nucleus, and staining in the cytoplasm and nucleus became stronger. In the LXA4 group, these changes were more obvious, and the activation of Nrf2 was significantly increased in the LXA4 group compared with that in the TNF-*α* group, indicating that the nuclear translocation of Nrf2 can be further promoted by LXA4. Next, immunoblot analysis (Figures 4(a) and 4(b)) showed that the expression of HO-1 was efficiently increased in the TNF-*α*+LXA4 group compared with that in both the control and TNF-*α* groups. We further tested relative luciferase activities in HPMECs. The results of the luciferase reporter assay in Figure 4(f) showed that Nrf2 more effectively regulated HO-1 than NF-*κ*B, AP-1, and NF-*κ*B combined with AP-1. We speculated that the Nrf2/HO-1 pathway may play a role in the protective effects of LXA4.

### 3.5. Inhibition of Nrf2 with a Lentivirus Attenuates the Anti-Inflammatory and Antioxidative Effects of LXA4 on HPMECs

**Figure 5:**
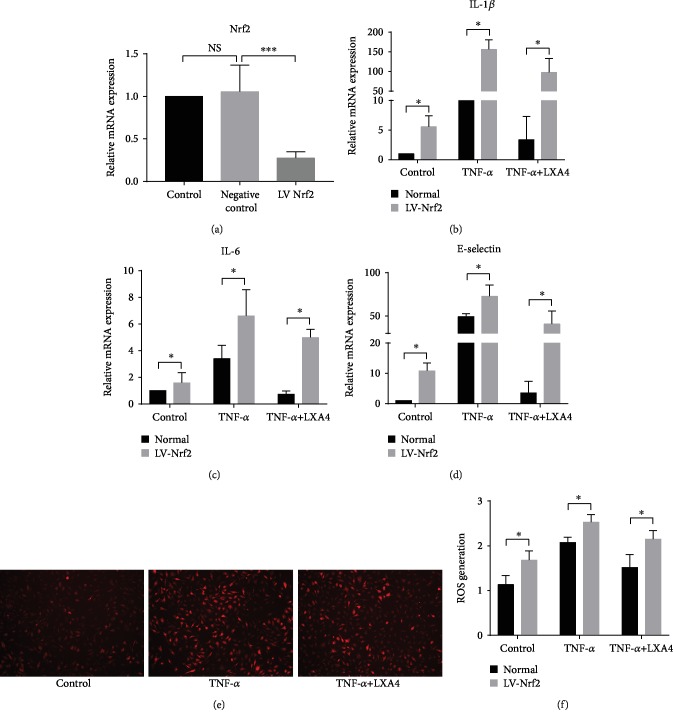
Knocking down Nrf2 expression attenuated the effects of LXA4 on the regulation of proinflammatory factors and ROS in HPMECs. (a) Nrf2 mRNA levels in HPMECs. The cells were transfected with a Nrf2 lentivirus for 72 h. The mRNA levels of Nrf2 were determined by RT-PCR. (b–d) Determination of IL-1*β*, IL-6, and E-selectin mRNA expression by RT-PCR. *β*-Actin served as an internal control. (e) Cellular ROS levels in the Nrf2 knockout HPMECs, measured using DHE. This experiment was independently repeated three times with 3 wells per time. (f) The quantitative mean fluorescence intensity in each treatment group is shown (^∗^*P* < 0.05, ^∗∗∗^*P* < 0.001). TNF-*α*: the TNF-*α* group; TNF-*α*+LXA4: the TNF-*α*+Lipoxin A4 group; LV Nrf2: the Nrf2 lentivirus-transfected group.

To further determine whether Nrf2 is involved in the beneficial effect of LXA4, we observed the effect of LXA4 on Nrf2-deficient cells. A specific lentivirus was used to knock down Nrf2 expression. The Nrf2 LV-treated group expressed lower levels of Nrf2 than the negative control and control groups (Figure 5(a)). As shown in Figures 5(b)–5(d) and 5(f), without stimulation, Nrf2 inhibition increased the expression of inflammatory factors and ROS in HPMECs. When HPMECs were challenged with TNF-*α* in the presence or absence of LXA4, Nrf2 LV-transfected cells generated higher IL-6, IL-1*β*, and E-selectin levels than normal cells. Similarly, ROS levels showed a considerable increase after either TNF-*α* or TNF-*α*+LXA4 stimulation compared with control treatment (Figures 5(e) and 5(f)). These results confirmed that LXA4 protects cells through Nrf2 activation.

### 3.6. Knocking Out Nrf2 Partially Abolishes the Protective Effect of LXA4 on Mice

**Figure 6:**
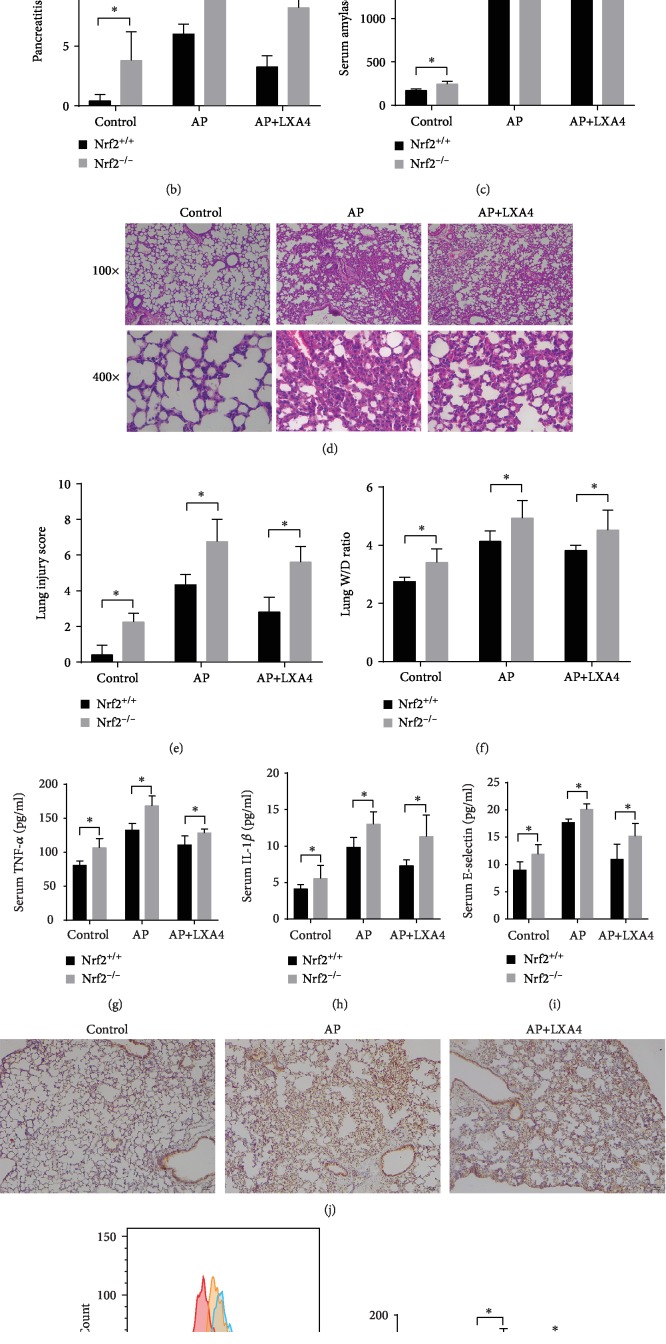
Knocking out Nrf2 partially abolished the anti-inflammatory and antioxidative effects of LXA4 on mice. Nrf2^−/−^ mice in the AP group were intraperitoneally injected with caerulein (50 *μ*g/kg, 7 times, ip) and LPS (10 mg/kg, ip), and those in the AP+LXA4 group were injected with LXA4 (0.1 mg/kg) before caerulein+LPS administration following the same protocol used for the Nrf2^+/+^ mice. (a, d) Representative morphological analysis of the pancreas and lung tissue of the Nrf2^−/−^ mice in the control, AP, and AP+LXA4 groups, (b, e) histological scores of the pancreas and lungs, (c) serum amylase levels, and (f) lung wet/dry weight ratios. (g–i) The levels of TNF-*α*, IL-1*β*, and E-selectin in the serum of mice measured by ELISA. (j) Images of IL-6 expression in lung tissue sections assessed by immunohistochemistry. (k) Representative images of the flow cytometric evaluation of cellular ROS levels in lung tissue from each treatment group. (l) Fluorescence signals detected using a flow cytometer. The mean fluorescence intensity values are graphed (^∗^*P* < 0.05). AP: the acute pancreatitis group; AP+LXA4: the acute pancreatitis+Lipoxin A4 group.

Nrf2^−/−^ mice were injected with or without caerulein+LPS or LXA4 following the same protocol used for Nrf2^+/+^ mice. The results in Figures 6(a)–6(c) showed that histological changes in the pancreas and serum amylase activity were largely increased in Nrf2^−/−^ mice compared with those in Nrf2^+/+^ mice in all groups. The results shown in Figures 6(d)–6(f) suggested that Nrf2^−/−^ mice had more obvious lung injury characteristics and damage than Nrf2^+/+^ mice. The protective effects of LXA4 on pancreatic and lung tissue damage in AP-ALI were reduced in Nrf2^−/−^ mice. Similarly, the decreased levels of proinflammatory cytokines, including TNF-*α*, IL-1*β*, and E-selectin, observed after LXA4 administration following AP were partially reversed in Nrf2^−/−^ mice (Figures 6(g)–6(i)). In addition, IHC staining for IL-6 in the lung tissues of Nrf2^−/−^ and WT mice (Figures 6(j)) and 2(d)) confirmed that Nrf2 mediates the effect of LXA4 on IL-6 expression. We next investigated whether the beneficial effect of LXA4 on AP-induced ROS production is Nrf2 dependent. Our results showed that the protective effect of LXA4 on AP-ALI was abolished in Nrf2^−/−^ mice (Figures 6(k) and 6(l)). Thus, the positive effect of LXA4 on AP-ALI may depend on Nrf2 activation.

## 4. Discussion

AP is the most common and devastating inflammatory disorder of the pancreas. AP remains a challenging clinical problem, and its detrimental effects can extend beyond the pancreas, resulting in local and systemic complications and a high mortality rate ranging from 20 to 30% in the most severe cases [30, 31]. The pathophysiology of AP is always considered in two phases. In the early stages, intrapancreatic activation of trypsin in acinar cells leads to autodigestion as well as the activation of various injurious inflammatory factors [32–34]. As AP progresses, acinar cell necrosis activates further pancreatic inflammation, which leads to SIRS or even organ damage, including ALI [32].

ALI, a common and severe complication of AP, usually occurs during the early phase and dramatically increases the risk of mortality associated with uncontrolled pancreatitis [35]. Many studies have attempted to identify effective therapies for AP, but this disease is still poorly treated outside of supportive care, highlighting the demand for new therapies targeting the underlying mechanism of AP.

LXA4 is an endogenous lipid mediator that displays an anti-inflammatory effect and has been extensively studied in a growing list of inflammation-related disease models [16], including fat embolism syndrome-induced lung injury [36], lipopolysaccharide-induced BV2 microglial activation and differentiation [16], and inflammation in diabetes-associated atherosclerosis [37]. Our group revealed that LXA4 can alleviate the symptoms of ALI associated with AP [38, 39]. However, the detailed mechanism by which this improvement occurs remains unclear.

AP-ALI is characterized by severe pulmonary edema and inflammation [38]. To determine the potential mechanism of the effect of LXA4, we employed HPMECs, the cells that form the interior barrier of the alveolocapillary membrane, as a convenient in vitro model to study [40]. Simultaneously, an experimental work in mouse models significantly increased our understanding of the pathophysiological links between AP and ALI. The present study indicated that histopathological changes, pathological injury scores, serum amylase levels, and lung W/D ratios in the AP group were significantly higher than those in the control group, which demonstrated that the mouse model of AP-ALI was successfully established. Mice pretreated with LXA4 exhibited obviously reduced serum amylase levels and lung W/D ratios and improved histopathological inflammatory damage in the lungs and pancreas. These results suggested that LXA4 can ameliorate the severity of AP and ALI.

In AP-ALI, the inflammatory response and inflammatory cytokines play pivotal roles and exert major influences on the outcome of the disease [5, 41]. The intricate balance of cytokine production relates to the degree of tissue damage [42]. Generally, lung edema due to the overexpression of inflammatory mediators is accepted as one of the major prerequisites for AP-ALI pathogenesis. HPMECs are attacked by inflammatory mediators, resulting in increased HPMEC permeability and barrier dysfunction [43]. After the alveolar barrier is damaged, a substantial amount of tissue fluid infiltration aggravates pulmonary edema, and the damaged lung function causes the blood oxygen saturation level to drop sharply, thus causing the body to be in a serious anoxic state and increasing the risk of mortality [6]. The well-known proinflammatory cytokines TNF-*α*, IL-1*β*, and IL-6 are considered pivotal promoters for initiating and perpetuating lung injury. TNF-*α* and IL-6 can recruit leukocytes into the lung tissue, while IL-1*β* can accelerate the process of lung damage by inducing monocytes and macrophages [44–46]. Meanwhile, E-selectin, which belongs to the selectin family of adhesion molecules, is expressed only on the surface of activated vascular endothelial cells, initiates the adhesion of leukocytes to vascular endothelial cells, and is increasingly important to judge the criticality and prognosis of ALI [47, 48]. In this study, our results confirmed that TNF-*α* treatment of HPMECs influenced the mRNA expression of IL-6, IL-1*β*, and E-selectin as measured by PCR and that these changes can be partly ameliorated in the presence of LXA4. Next, an ELISA demonstrated that LXA4 significantly reduced the serum TNF-*α*, IL-1*β* and E-selectin levels that were increased in the mice in the AP group. In addition, the IL-6 level in the lung tissue was increased in the mice in the AP group but mitigated by LXA4 pretreatment. LXA4 efficiently inhibited proinflammatory cytokine release to attenuate local and systemic inflammatory responses.

Recent research indicates that disruption of the balance between oxidation and reduction is believed to play a vital role in the occurrence and development of ALI [49, 50], especially in early AP-ALI. Excessive amounts of ROS can be produced by activated neutrophils, resulting in severe oxidative stress damage and irreversible injury to tissues and cells [51, 52]. Reductions in the production of the important oxidants of ROS are protective against AP-ALI. We frequently noticed that LXA4 can inhibit ROS generation. On this basis, we explored the influence of LXA4 on oxidative stress in vitro and in vivo. Similar to others' previous work [53], very low ROS levels could be detected in normal mice and cells. As popularly accepted, ROS production was enhanced in the mice in the AP group. This finding was in line with our expectation that HPMECs would also produce ROS locally after TNF-*α* stimulation. In our study, LXA4 produced an excellent effect to block AP-triggered ROS accumulation in lung tissue, which agreed with the result in HPMECs.

Nrf2, which is responsible for the regulation of the cellular redox balance and protective antioxidants, has emerged as a therapeutic target for oxidative stress [54–56]. Activating Nrf2 is a crucial strategy for inhibiting ROS generation and controlling oxidative stress. In addition, Nrf2 is known to be an important regulator in ALI [57–59]. Our results showed that TNF-*α* stimulation upregulated Nrf2 expression, and LXA4 further enhanced Nrf2 activation and decreased injury in HPMECs. Normally, Nrf2 is located in the cytoplasm and translocates into the nucleus after stimulation. Evidence from Western blotting and cellular immunofluorescence in our current investigation showed that the total and nuclear Nrf2 protein levels markedly improved after LXA4 treatment. Moreover, TNF-*α*-induced phosphorylation of Ser40 in Nrf2 was further enhanced by LXA4. Our results showed that LXA4 increased the Nrf2 level in the nucleus and the phosphorylation of Ser40 in Nrf2.

Nrf2 binds to AREs in the nucleus and enhances the expression of downstream target genes, including HO-1 [60]. HO-1 is a cytoprotective antioxidant enzyme that plays a protective role under both physiological and pathological conditions [61]. The present results agree with those for Nrf2; LXA4 has the ability to switch the activity of HO-1 in TNF-*α*-treated HPMECs from a high expression state to a much higher expression state. The mechanism by which LXA4 activates HO-1 remains to be further studied. A luciferase assay was employed to show the major role of Nrf2 in the activation of HO-1 by recording the fluorescence intensity of HPMECs transfected with NF-*κ*B, AP-1, and Nrf2.

Moreover, to determine the role of the Nrf2 signaling pathway in the LXA4-mediated mitigation of AP-ALI, HPMECs were transfected with a Nrf2 gene knockout lentivirus. Here, IL-1*β*, IL-6, and E-selectin mRNA levels were higher in the Nrf2-knockout HPMECs than those in normal cells in all three groups. In TNF-*α*-stimulated Nrf2-knockout cells, ROS production was significantly increased, and the preventive effect of LXA4 was not obvious, suggesting that LXA4 displays Nrf2-dependent antioxidant activity. To further assess the function of Nrf2 in the antioxidant response, we compared Nrf2^−/−^ mice with WT mice. Pathological damage was compared in the pancreas and lungs of WT and Nrf2^−/−^ mice after modeling and treatment, and more intense inflammatory cell infiltration, edema, and even necrosis were found, as expected, in the Nrf2^−/−^ mice compared to observations in the WT mice. Mice with AP-ALI also showed increasing *W*/*D* ratios, which were greater in Nrf2^−/−^ mouse lungs than those in WT mouse lungs. In turn, higher levels of inflammatory cytokines were observed in the Nrf2^−/−^ mice than those in the WT mice. A decrease in ROS production was observed in WT mice pretreated with LXA4 compared with WT mice in the AP group. Furthermore, a higher increase in ROS production was observed in the Nrf2^−/−^ mice than that in the WT mice under every condition.

In summary, our study showed that LXA4 played a protective role against AP-ALI and exhibited anti-inflammatory properties in TNF-*α*-stimulated HPMECs. LXA4 also displayed significant antioxidant effects, thus reducing ROS levels. LXA4 attenuated AP-ALI, which was mediated at least in part by the Nrf2/HO-1 pathway. Of course, some limitations exist, and further studies are needed to clarify the mechanism in greater detail.

## Figures and Tables

**Table 1:** Sequences of the primers used for quantitative real-time PCR.

Gene	Forward primer (5′–3′)	Reverse primer (5′–3′)
*β*-Actin	CCTGGCACCCAGCACAAT	GGGCCGGACTCGTCATAC
IL-1*β*	GCGGCATCCAGCTACGAATCTC	AACCAGCATCTTCCTCAGCTTGTC
IL-6	CCTCCAGAACAGATTTGAGAGTAGT	GGGTCAGGGGTGGTTATTGC
E-selectin	GAAGAGGTTCCTTCCTGCCAAGTG	CAGAGCCATTGAGCGTCCATCC
Nrf2	ACCTCCCTGTTGTTGACTT	CACTTTATTCTTACCCCTCCT

## Data Availability

The data used to support the findings of this study are included within the article.
